# Secondary (Late) Developmental Dysplasia of the Hip with Displacement: From Case Studies to a Proposition for a Modified Diagnostic Path

**DOI:** 10.3390/diagnostics12061472

**Published:** 2022-06-15

**Authors:** Jacek Dygut, Jerzy Sułko, Ibeth Guevara-Lora, Monika Piwowar

**Affiliations:** 1 Independent Public Healthcare Center in Międzyrzec Podlaski, Warszawska 2/4 st., 21-560 Międzyrzec Podlaski, Poland; jacek.dygut@gmail.com; 2 Wojewódzki Specjalistyczny Szpital Dziecięcy im Św. Ludwika w Krakowie, Strzelecka 2 st., 31-503 Krakow, Poland; jerzysulko@hotmail.com; 3 Department of Analytical Biochemistry, Faculty of Biochemistry, Biophysics and Biotechnology, Jagiellonian University, 31-007 Kraków, Poland; ibeth.guevara-lora@uj.edu.pl; 4 Department of Bioinformatics and Telemedicine, Faculty of Medicine, Jagiellonian University Medical College, 7e Kopernika st., 31-034 Krakow, Poland

**Keywords:** a case report of developmental dysplasia of the hip, diagnosis of dysplasia, treatment standards for developmental dysplasia of the hip

## Abstract

(1) Background. This paper presents a case of hip joints that were initially described as either normal or physiologically immature in four successive ultrasound examinations using the static method by Graf; however, the final treatment of the patient involved multiple hip reconstruction surgeries. (2) Case presentation. The patient was born with an Apgar score of 10 and did not exhibit neurological diseases that could deform and lead to pathological dislocation of the right hip joint. The subsequent analysis of medical data revealed that the hip luxation was due to secondary (late) developmental dysplasia of the right hip. (3) Conclusion. The article emphasizes the importance of early diagnosis and treatment standards for developmental dysplasia of the hip (DDH). The development of uniform international medical guidelines for the diagnosis, treatment, and prevention of hip dysplasia, along with the unification of DDH-related terminology, would allow for more effective management of DDH cases and reduce the cost of patient treatment.

## 1. Introduction

Developmental dysplasia of the hip (DDH) in newborns is quite frequently diagnosed. DDH affects 1–2% of newborns, depending on whether the clinical or ultrasound definition is used [1,2]. In populations subjected to Ortolani and Barlow clinical tests (when there is either dislocation or a predisposition toward dislocation) the incidence ratio is 1.6–28.5 per 1000 newborns [3].

Research indicates several risk factors [4]. This disorder is known to occur five times more frequently in girls, and in about 20% of cases, a similar defect is also present in a member of the immediate family, i.e., siblings, parents, or grandparents. Furthermore, it has been observed that there are regions of the world where hip dysplasia is more common than elsewhere [5].

This problem is widely discussed in the scientific literature [6,7]; however, despite numerous studies, the issue is not fully understood and sufficiently described [8]. Specialist literature tends to be nonuniform when it comes to hip dysplasia-related terminology, with most publications using the following terms interchangeably: “congenital hip dysplasia” [8], “congenital hip dislocation” [9], and “developmental hip dysplasia” [10]. According to the International Classification of Diseases and Health Problems (Q65 to Q79) the term “congenital hip dislocation” still applies [11], although “developmental hip dysplasia” (DDH) is becoming increasingly common. DDH is described as a set of disorders within the hip joint that emerge during development, manifest in various ways and at different ages, and are not always detectable at birth. [12] More specifically, it has been estimated that about 15% of DDH cases are undetectable at birth even by experienced physicians and ultrasound examination specialists [13]. The term DDH describes a broad spectrum of abnormalities, from instability observed in infants (subluxation or dislocation) to hip dysplasia (flattening or hypoplasia of the acetabulum) [14]. The exact definition of hip dysplasia is still somewhat controversial: there is no agreement on the specific set of causes and symptoms that constitute pathological dysplasia as well as on precise treatment criteria [15].

The definition of DDH assumes that developmental hip dysplasia may occur in the embryonic period (up until the 10th week of pregnancy), although it is known that the fissure secretes the future femoral head and acetabulum develops only in the 7th week. Consequently, this is the moment when the onset of DDH can be theoretically considered [12,16]. In practical terms, the earliest point at which hip dislocation may occur is the 11th week of fetal life, i.e., when the hip joint becomes fully formed. In contrast to DDH, all hip dislocations resulting from organogenesis disorders are called teratogenic intrauterine dislocations. In the case of developmental dysplasia of the hip, anatomical structures of the joint, normal in the embryonic period, gradually become abnormal due to the action of a persistent deforming factor [12]. As a result, the tissue structure is disrupted, qualifying the disorder as dysplasia rather than deformation. The precise alignment of joint elements lost in DDH and the femoral head may have a predisposition to slide out of the acetabulum [16]. In the absence of correct diagnosis and proper treatment after birth, the natural progression of developmental dysplasia of the hip may unfold in one of four scenarios [16]:

- The hip may become normal (spontaneous healing);
- The hip may progress to subluxation (partial contact of joint elements);
- The hip may progress to luxation (dislocation);
- The hip may remain in place, but with dysplastic features.

The case presented in the paper suggests the need to revise and clarify the applicable nomenclature to avoid misinterpretation and misunderstanding of the etiology of DDH. Considering the time of the occurrence of the defect, the following division of hip dysplasia was suggested:

Prenatal period: the primary (early) developmental hip dysplasia caused by genetic and/or biochemical and/or biophysical and/or mechanical factors in the fetal period.

Postnatal period: secondary (late) developmental dysplasia of the hip caused by genetic and/or biochemical and/or biophysical and/or mechanical and/or iatrogenic factors in the postnatal period.

The paper describes a case of secondary (late) developmental hip dysplasia in which the mechanical damaging factor, i.e. contracture in adduction, gradually forcing out the right femoral head, occurred after the birth of the child. No disorders in the anatomical structure of the hip joints were observed in the neonatal period or early infancy (confirmed by four USG examinations), although the result of a physical examination indicated a functional disorder that could potentially result in a deepening pathology as the child developed. In this instance, a healthy, undeformed hip joint in a newborn (and subsequently in an infant) was affected by a long-term extraarticular factor gradually deforming it. In terms of pathomechanics, similar cases have been described in publications in which the main deforming factor (forcing the head out of the hip joint) was active after the birth of the child [4,7,17].

A similar mechanism based on forcing out the femoral head can result from poor childcare, which includes wrapping the lower limbs of newborns and infants in an extended position rather than in the “spike” position, carrying the child in a face-forward position, improper child placement (only on one side), etc. In most cases, however, no diagnostics were performed to check whether the hip joint was normal after birth or whether it already exhibited dysplastic changes in the prenatal period, with poor infant care (secondary iatrogenic factor) only exacerbating a preexisting disorder and resulting in full hip dislocation.

In summary, in the assessment of the abnormal hip development, the greatest challenge involves confusion between dysplasia and deformity, which often leads to misdiagnosis, incorrect nomenclature, and incorrectly chosen treatment. Moreover, existing standards do not provide explicit methods along with a full range of diagnostic options.

The case presented in the paper clearly shows this problem. It is an example of secondary (late) Developmental Dysplasia of the Hip (late DDH). It is distinguished by the fact that the course of DDH ended with total dislocation of the joint regarded as healthy at birth (from the point of view of its anatomical structure), even though in terms of function, it was a high-risk hip because bilateral postpartum contracture had been noted. The message of the case report is to draw attention to the possibility of abnormal development of hip joint tissues in the late postnatal period; it seems necessary to perform control examinations within 6 and 12 months after birth.

## 2. Materials and Methods

The medical history of the case of a child with DDH, including a description of medical examinations and accompanying examinations, was obtained from court files. A detailed step-by-step analysis of the treatment procedure was performed (from 2006 to 2010). As the data come from paper court documents and not from the hospital IT system, the quality of the scans is not very good. However, due to the uniqueness of the material, it was decided to include it in this format of the manuscript.

A literature review from the Pubmed database was carried out in the subject area. Based on the patient’s data and the results of medical examinations and accompanying examinations, as well as a review of the treatment regimens used so far, a therapeutic path for DDH was proposed.

## 3. Case Presentation and Results

Upon birth, the child (girl) scored 10 on the Apgar scale. Four subsequent ultrasound examinations (at 5, 9, 13, and 16 weeks of age) described hip joints as either normal or physiologically immature (according to Graf). Physical examination revealed an evident pathognomonic functional disorder characteristic for dysplasia, manifested by the adduction contracture of both hips. Conservative treatment with an abduction apparatus, necessary in this case, was abandoned, which ultimately resulted in multiple reconstructive surgeries of the right hip joint.

The girl did not exhibit neurological disorders such as cerebral palsy, myelomeningocele, or arthritis that could lead to secondary deformation and pathological dislocation of the hip joints. Basic genetic defects were excluded, as was the most important individual risk factor—breech position. Following delivery, the attending pediatrician at the Neonatology Department recognized “asymmetry in the abduction of the hip joints” and referred the patient to a preluxation clinic as well as to an orthopedic specialist. Faced with a long (three-month) waiting period, the child’s parents went to a private ultrasound laboratory, where the same doctor treated the child again (the doctor was also employed at the preluxation clinic).

**Figure 1:**
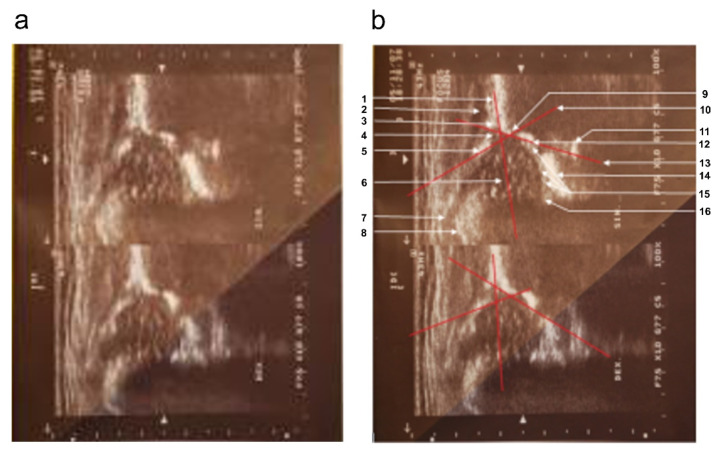
Sonograms of the girl’s hip joints at 5 weeks of age (first appointment). (**a**) Top (left hip joint): three basic elements are visible, i.e., the labrum, the “blunt” bone edge marking the point where the bulge of the iliac bone passes into the concavity of the acetabulum, and the lower edge of the iliac bone. The alpha angle between the bone roofline and the baseline is 65°; the beta angle between the cartilaginous roofline and the baseline is 69°. Lack of ossification nuclei (physiological norm). Type Ib according to Graf. Bottom part (right hip joint): three basic elements are visible: the labrum, the “rounded” bone edge marking the point where the protrusion of the iliac bone passes into the concavity of the acetabulum, and the lower edge of the iliac bone. The alpha angle between the bone roofline and the baseline is 56°; the beta angle between the cartilage roofline and the baseline is 74°. Lack of ossification nuclei (physiological norm). Type IIa according to Graf. (**b**) Sonograms with overlay and description of anatomically important points. Top: 1—baseline, 2—gluteus minimus muscle, 3—tendon of the femoral rectus muscle, 4—the cartilaginous roof of the acetabulum, 5—labrum, 6—cartilaginous femoral head (no ossification nucleus), 7—cartilaginous major trochanter with the turning synovial fold of the joint capsule, 8—cartilage–bone boundary, 9—bone edge (blunt type), i.e., turning point, 10—cartilage roofline, 11—internal perichondrium of the “Y” cartilage, 12—lower ilium bone edge, 13—bone roofline, 14—soft tissues at the bottom of the acetabulum, 15—round ligament of the femoral head, 16—transverse ligament. **Bottom:** marked baseline, cartilage roofline, and bone roofline.

The first time the child underwent an ultrasound examination was 5 weeks after birth. During the first appointment, a visiting pediatric surgeon presented the following description: “Outlines of acetabular roofs in both hip joints slightly steepened, shallow and flattened. Acetabulum roofs on both sides are slightly blunted. The cartilaginous labrum in both hips is unremarkable. Ossification nuclei of femoral heads not yet outlined (age norm)” (Figure 1). In the description of the ultrasound, the doctor did not provide specific measurements of the alpha and beta angles.

**Figure 2:**
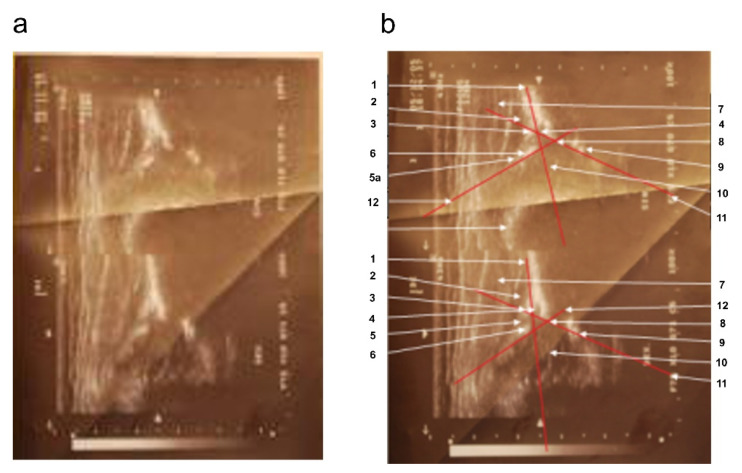
Sonograms of the girl’s hip joints at 9 weeks of age (second appointment). (**a**) Top part: left hip joint; alpha angle: 53°; beta angle: 74°; round bone edge. Type IIa according to Graf. Bottom: right hip joint; alpha angle: 63°; beta angle: 62°; acute bone edge type. Type Ib according to Graf. (**b**) Sonograms with overlay and description of anatomically important points. 1—baseline, 2—gluteus minimus muscle, 3—tendon of the femoral rectus muscle, 4—cartilage acetabular roof, 5—the disappearance of the joint capsule shadow in the region of the perichondrium defect in the adipose cushion; 5a—articular capsule, 6—acetabular labrum, 7—gluteus medium muscle, 8—bone edge (blunt type), i.e., turning point, 9—lower edge of the iliac bone, 10—cartilaginous femoral head with visualized vascular sinuses, 11—bone roofline, 12—cartilage roofline.

Based on subsequent analysis of the progress of the disease (ultrasound examination scans), the left hip joint was assigned to type Ib and the right hip joint to type IIa according to Graf. The description of a sonogram performed at the same lab during the second visit (4 weeks later), at 9 weeks of age, emphasizes: “better visibility of the contours of both bone roofs, both acetabula less steep, deeper, more arched. The roofs of both acetabula are fairly sharp (correct). Cartilage labrum is good. Ossification nuclei of heads not yet outlined (age norm). Alpha and beta angles within normal ranges” (Figure 2). 

The third visit was performed at 13 weeks of age (i.e., 4 weeks later, consistent with the aforementioned 3-month waiting period) at the state preluxation clinic where the doctor in charge of the patient worked. Here, for the first time, the results of a physical examination were recorded indicating a slight reduction in the abduction of both hip joints with the annotation “hard hips”. The description of the ultrasound examination indicated the presence of somewhat steep and shallow acetabula and a slightly flattened left acetabulum. The mother was instructed about the need for intensive abduction prevention by using double (safety) diapers.

During the 4th visit (in the 16th week of life) at a private ultrasound laboratory with the same attending physician, the presence of correctly centralized ossification nuclei of both femoral heads was noted for the first time (more evident on the right side, with bilaterally normal (acute) acetabular roofs and cartilage labrum of the acetabulum. Alpha and beta angles in both hip joints were within normal limits. Based on ultrasound scans, both hips were assigned to group Ia° according to Graf. The fourth visit concluded with the diagnosis and observation of the patient, without further follow-up recommendations.

**Figure 3:**
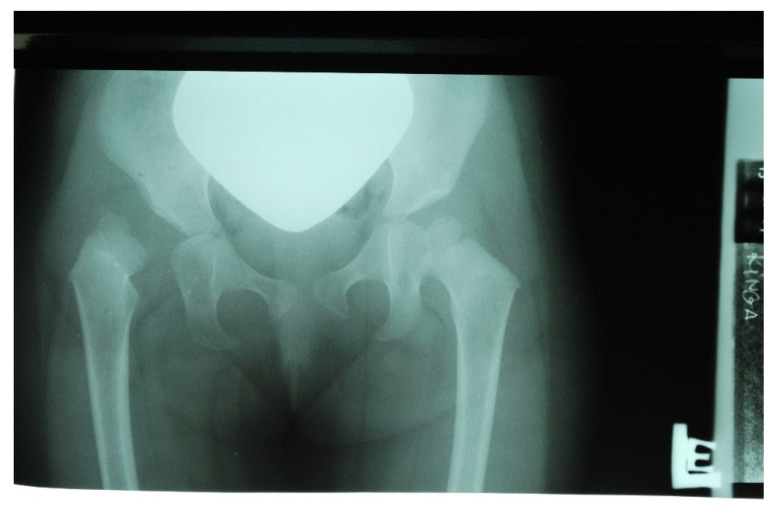
X-ray scan showing right hip dislocation in the girl (at 2 years of age).

Over time, the parents noticed that the child was very slow to develop motor skills: sitting down and walking. The child did not learn to walk until 22 months of age. While climbing on a slide, the girl always used her lower left limb, without alternating her gait. While walking, she limped on her right lower limb, and reported “right leg pain”. With these observations, the parents again reported to the attending physician, an employee of a private ultrasound lab and national preluxation clinic. Due to the child’s age (24 months), an X-ray of the pelvis was performed, indicating dislocation of the right hip joint (Figure 3).

Following orthopedic consultation, the girl qualified for surgical treatment (reconstruction of the right hip joint with corrective rotational femoral osteotomy on the right side).

**Figure 4:**
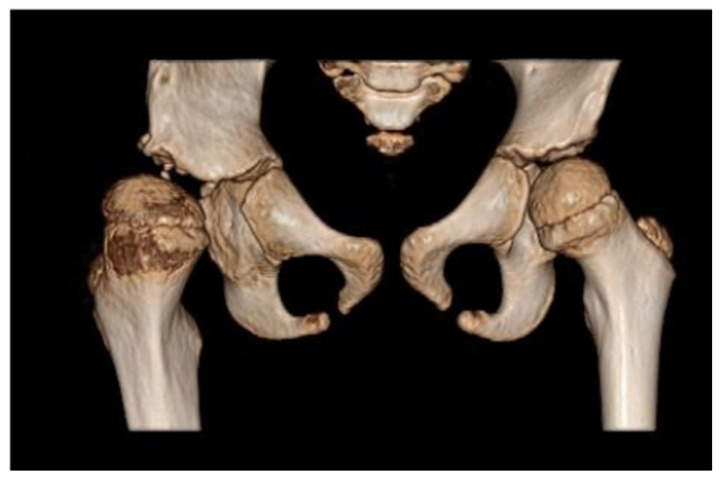
Computed tomography (CT) of the pelvis—3D scan carried out at 5 years of age. The relative tilt angle of the femoral head is about 47°; the angle of internal rotation is about 25°. Knee set in internal rotation. Femoral head in anterolateral subluxation. Joint space significantly widened. Thickening of soft tissues around the hip joint; exudate cannot be excluded. Differentiation reveals postoperative changes and inflammation. The acetabulum is slightly distorted in the area of the roof and flattened with irregular contours, marginal sclerotization, and small defects. A ~4 mm calcification or ossification can be seen in the soft tissues in front of the anterior part of the acetabular roof and below the posterior edge of the roof. The femoral head is flattened. There is marked thinning of the bone structure of the femoral head and neck.

**Figure 5:**
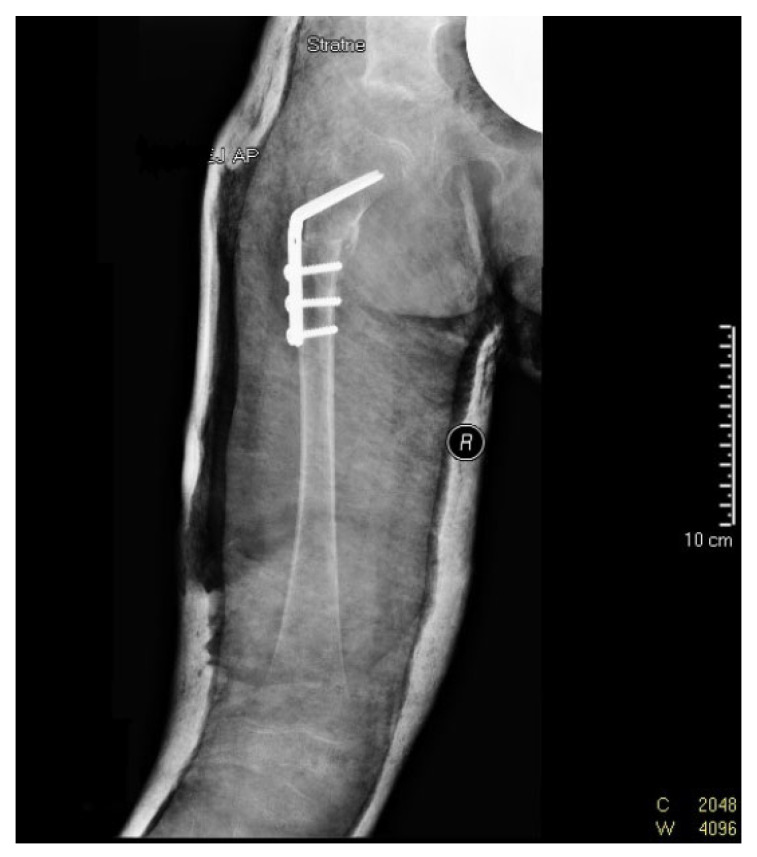
X-ray scan following varus and de-rotative osteotomy on the proximal part of the right femoral bone (at 6 years of age).

Due to persistent radiological evidence of subluxation in the right hip joint, at the age of 4.5, another pelvis osteotomy was performed using the Salter method. At the age of five, all metallic osteosyntheses were surgically removed and a pelvic computed tomography (CT) scan was performed (Figure 4). The next (third) surgery involving varus direction and de-rotative osteotomy on the proximal part of the right femur was performed on the girl at age 6 (Figure 5).

Ultrasound scans from four follow-up visits were evaluated several times by court experts who found their performance and description correct. Each of the scans met the evaluation standards set by Graf and confirmed the correct anatomical structure of the patient’s hip. Four independent ultrasound examinations ruled out the possibility of malfunctioning ultrasound equipment or errors in the description.

The analysis of medical data reveals that the root cause of pathological changes observed in the girl was an external factor, namely abduction contracture of the right hip joint. This caused incorrect positioning of the lower limb (which had previously been developing properly), forcing incorrect development of the right hip joint during the child’s growth and ultimately leading to luxation. Finally, the child was diagnosed with secondary (late) dislocation of the hip in the course of dysplasia.

## 4. Discussion

In the extensive field of diagnostics and the treatment of developmental hip dysplasia (DDH), no uniform diagnostic or therapeutic standards are recognized around the world. This problem includes the frequency and complementarity of medical examinations, especially in the first weeks of a child’s life [10,12,18,19,20].

In many publications, attention is drawn to prevention, starting at birth, and then to protection and remediation of congenital or acquired developmental abnormalities [14,16]. In Anglo-Saxon countries, the basis for diagnosis is a physical examination conducted after birth and focused mainly on the presence of Barlow and Ortolani signs (up until the end of the second month). At three months of age, Barlow and Ortolani signs turn negative, and the restriction of abduction (as well as abduction asymmetry) emerges as the most reliable DDH diagnostic criterion [3,10,21].

The search for pathognomonic symptoms in a physical examination is consistent with evidence-based recommendations developed by the American Academy of Orthopedic Surgeons (AAOS) for the assessment of hip states up until the sixth month of life [22].

Physical examination is regarded as the basic screening test. Ultrasound examination is recommended if disorders are found in a clinical examination at 6 weeks of age, and also in the presence of risk factors, i.e., positive family history (DDH in parents and/or siblings) or breech position. The latter is, in fact, the most significant risk factor for the development of secondary dysplasia, and even in children with correct clinical examination results and unremarkable ultrasound scans, X-rays of the hip should be performed at 6 months of age [22].

The protocol where a physical examination is conducted first, while additional tests (ultrasound, X-ray) follow only if a disorder is detected, may prove insufficient in certain cases. It risks overlooking the so-called **clinically mute dysplasia**, which applies to about 6% of cases [14,16]. DDH is an evolving process, and therefore physical examinations should change as the child grows older. In particular, normal physical examination findings during the immediate postnatal period do not preclude a subsequent diagnosis of DDH [10]. There are reports of otherwise healthy children with normal physical examinations and radiographs of the hip in the first 3 months of life who later developed hip dislocation [23].

In the case described in the paper, numerous ultrasound check-ups carried out after childbirth indicated that the anatomical parameters of the joint were consistent with normal limits, while a physical examination revealed functional abnormalities of the hip.

Similar to clinically mute dysplasia, this type of dysfunction can be defined as **diagnostically mute dysplasia.** It shows that even an unremarkable ultrasound image of the child’s hips (so-called Graf type I or IIa) does not guarantee the correct development of the joint.

The comprehensive assessment of the hip joint is not limited to anatomy or joint structure (visualized on ultrasound or X-ray scans) but must also include joint function and range of hip motion. The joint can be anatomically normal but functionally incorrect, due to, for example, the limitation of hip abduction. Inactivity in the face of asymmetrical hip abduction can lead to secondary (late) developmental hip dysplasia with dislocation [10].

Therefore, even when ultrasound results do not indicate any abnormality, the doctor may nevertheless recommend treatment. This should not be regarded as going against the accepted methodology for treating dysplasia.

In this context, it is important to note that should any irregularities be found in the neonatal or infant period and recorded by the pediatrician, pediatric surgeon, or radiologist, the decision about further treatment should be made by an orthopedic specialist. The orthopedist, possessing the most extensive knowledge of the child’s hip physiology, should not neglect a dynamic test, which is crucial for determining the stability of the hip, and should recommend follow-up check-ups if necessary [10].

In the United Kingdom, the Standing Medical Advisory Committee (SMAC) publishes updated guidelines for DDH screening. At present, they state that all children should undergo a clinical examination on the first day after birth, before discharge, during the sixth week of life, between the sixth and the ninth month of life, and after they start walking [15]. Similar recommendations are made in other countries [23].

In the described case, upon detecting the restricted abduction of both hip joints, it was necessary to administer treatment using Pawlik’s harness, Frejka’s pillow, or Koszla splint, regardless of the results of the ultrasound examination. The implementation of sham treatment involving safety double diapers should be regarded as incorrect and insufficient.

At this point, it should be noted that sometimes a situation may emerge where the diagnostic procedures (scans) produce results similar to those presented in this publication, but the associated etiology turns out to be different. This may occur when there are small differences in the sequence of genes that are important from the point of view of connective tissue function (e.g., genetic variabilities headed by single nucleotide polymorphisms (SNP)) modulating the functions of proteins but also other biomolecules, e.g., regulatory RNA [24,25]. In such cases, the doctor may not observe pathological anatomical changes in the hip in the first weeks of the child’s life. However, such genetic variations can have a long-term impact on tissue function and disrupt the anatomical development of the acetabulum, eventually leading to secondary (late) dysplasia with functional disorders of the extra-articular tissues (contracture, excessive flaccidity) [26,27]. This is especially true in the first eight years of life as the acetabulum undergoes formation [16]. These changes may further lead to joint discongruity (mismatch) and progress to hip dislocation. The same genetic factors are likely involved in the so-called recurrent dysplasia [10].

Considering the above issues, it should be noted that neonatal hip examinations must acknowledge the risk of clinically and diagnostically mute cases given that genetic diagnostics is not yet a routine procedure.

Therefore, it is important to augment physical examination with complementary diagnostic (including genetic) tests. Overlooking a defect may have a substantial impact on the quality of the patient’s life in the future [28]. This is, in fact, one of the main causes of childhood and adulthood disability, whose treatment imposes a serious burden on public budgets [15].

Due to the lack of uniform international clinical management standards, as well as uniform terminology used to describe hip dysplasia, one can encounter differing diagnostic and clinical approaches along with imprecise descriptions. This makes it difficult to precisely define the correct procedures to be followed by doctors and to determine the validity of the chosen diagnostic and therapeutic path. In light of these difficulties, cases involving alleged medical malpractice become particularly difficult to adjudicate.

The paper presents a case of secondary (late) hip dysplasia that shows that the typical approach may result in an incorrect assessment of the patient’s health and, as a consequence, lead to disability. In the described case, abduction contracture, whose detection and evaluation is possible only by way of physical examination, progressively deformed an initially healthy hip joint, eventually causing its dislocation.

In the presented case, hip joint contracture was not a clinical, i.e., a follow-up (secondary) symptom of dysplasia but its primary cause. Contracture may emerge as an independent disease associated with the spontaneous fibrosis of the adductor muscles or a temporarily disturbed muscular balance between synergists and antagonists (adductors and abductors). In this case, transient neurogenic perinatal dystonia also cannot be excluded. The anatomical changes that arose in the hip joint should be described as developmental late hip deformity. This is strictly in line with the definition of deformation (Latin: *deformatio*) in which a properly developed anatomical structure is deformed by persistent mechanical factors. The original deformation involved the acetabulum, while changes in angles and the overall shape of the proximal femoral epiphyseal were a secondary process.

## 5. Conclusions

With this approach, the chances of detecting both early and late (3 months after birth) dysplasia would increase significantly. Despite various screening programs, the number of DDH cases has not decreased over the years, and the surgery rate has increased [29,30]. For this reason, in 2000, the American Academy of Pediatrics developed further guidelines for DDH research based on literature data and expert opinions [31]. In 2014, American Academy of Orthopedic Surgeons (AAOS) experts developed a set of rigorous evidence-based clinical practice guidelines to raise DDH detection standards [22].

AAOS guidelines are effective in the presence of diagnostically mute dysplasia when functional disorders (adduction contracture) occur with normal hip anatomy demonstrated on ultrasound or X-ray. In such cases, the result of the physical examination determines the implementation of the therapeutic (abduction) procedure, while ultrasound and X-ray examinations play a supportive role.

However, the problem remains open because existing guidelines do not guarantee the detection of clinically mute dysplasia, in which there are no pathognomonic symptoms of hip instability or limited abduction, while dysplastic features are only detectable on ultrasound or X-ray scans.

The development of uniform international medical guidelines for the diagnosis, treatment, and prevention of hip dysplasia, along with the unification of DDH-related terminology, would allow for more effective management of DDH cases and thus significantly reduce the cost of patient treatment.

The main message of this DDH case study is that physical examinations and additional examinations, such as diagnostic tests (USG, RTG, genetic), should be routinely performed in the screening procedure to identify both clinically and diagnostically mute cases.

The clarification of the diagnostic scheme could significantly affect the course of treatment in newborns but also in older patients (late dysplasia). Doctors with knowledge of genetic variations that affect joint tissues, and possible scenarios for the development of pathological changes, could plan preventive procedures to mitigate the consequences of severe degenerative changes observed in the third and fourth decades of life [13,32].
